# VV-ECMO in critical COVID-19 obese patients: a cohort study

**DOI:** 10.1186/s44158-024-00191-1

**Published:** 2024-08-13

**Authors:** Joana Nogueira, Ricardo Freitas, José Eduardo Sousa, Luís Linhares Santos

**Affiliations:** Intensive Care Medicine, Coimbra University Hospital Centre, Coimbra, Portugal

**Keywords:** VV-ECMO, Obesity; COVID-19

## Abstract

**Background:**

Obesity causes significant difficulties in successful extracorporeal membrane oxygenation (ECMO) support and may interfere with patient outcomes. During the COVID-19 pandemic, we experienced an increased number of obese patients supported with ECMO in our intensive care unit due to severe illness in this population.

**Methods:**

We designed a single-center retrospective study to identify prognostic factors for 180-day survival in obese critical COVID-19 patients receiving venovenous ECMO (VV-ECMO). We included adult critical COVID-19 patients on VV-ECMO, who were obese and overweight (according to the World Health Organization) and admitted to a tertiary hospital’s intensive care unit from April 1, 2020, to May 31, 2022. Univariate logistic regression analysis was performed to assess differences in 180-day mortality.

**Results:**

Forty-one patients were included. The median age was 55 (IQR 45–60) years, and 70.7% of the patients were male. The median body mass index (BMI) was 36 (IQR 31–42.5) kg/m^2^; 39% of patients had a BMI ≥ 40 kg/m^2^. The participants had 3 (IQR 1.5–4) days of mechanical ventilation prior to ECMO, and 63.4% were weaned from VV-ECMO support after a median of 19 (IQR 10–34) days. The median ICU length of stay was 31.9 (IQR 17.5–44.5) days. The duration of mechanical ventilation was 30 (IQR 19–49.5) days. The 180-day mortality rate was 41.5%. Univariate logistic regression analysis revealed that a higher BMI was associated with greater 180-day survival (OR 1.157 [1.038–1.291], *p* = 0.009). Younger age, female sex, less invasive ventilation time before ECMO, and fewer complications at the time of ECMO cannulation were associated with greater 180-day survival [OR 0.858 (0.774–0.953), *p* 0.004; OR 0.074 (0.008–0.650), *p* 0.019; OR 0.612 (0.401–0.933), *p* 0.022; OR 0.13 (0.03–0.740), *p* 0.022), respectively].

**Conclusion:**

In this retrospective cohort of critical COVID-19 obese adult patients supported by VV-ECMO, a higher BMI, younger age, and female sex were associated with greater 180-day survival. A shorter invasive ventilation time before ECMO and fewer complications at ECMO cannulation were also associated with increased survival.

## Background

Approximately 20% of the intensive care unit (ICU) population has a body mass index (BMI) ≥ 30 kg/m^2^ [1, 2]. It is known that obese patients are prone to more comorbidities and changes in respiratory mechanisms resulting in hypoxemia and hypercapnia [3]. They also have an increased risk of acute respiratory distress syndrome (ARDS) in the intensive care unit (ICU) setting [4].

In severe ARDS, extracorporeal membrane oxygenation (ECMO) can be indicated as it decreases mortality [5, 6]. Although potentially creating difficulties for the extracorporeal technique, obesity is not a contraindication for ECMO support [7]. However, this condition poses significant challenges to physicians and may interfere with patient outcomes. The main difficulties in these patients are safe vascular cannulation and obtaining adequate blood flow to meet the patient’s demands [8]. A greater inflammatory state also increases susceptibility to hypercoagulability, with possible thrombotic complications or the need for higher doses of anticoagulation [8].

During the COVID-19 pandemic, we experienced an increased number of obese patients supported with ECMO in our ICU due to severe illness in this population. In our research, we sought to understand which obese patients may benefit the most from this extracorporeal technique.

## Methods

### Study design and setting

We designed a retrospective study to identify prognostic factors for 180-day survival in critical COVID-19 obese patients receiving venovenous ECMO (VV-ECMO). This single-center retrospective study enrolled adult patients with COVID-19 associated pneumonia who were obese or overweight and were supported with VV-ECMO for severe ARDS. Patients were admitted to our tertiary hospital’s ICU at Coimbra Hospital and University Centre.

COVID-19 associated pneumonia was diagnosed by the presence of new or worsened radiological infiltrates associated with clinical or laboratory finding suggestive of infection: a temperature of over 38 °C or under 36 °C, purulent respiratory secretions and a leucocyte count of over 10.000/mm^3^ or leukopenia under 4.000/mm^3^. Severe acute respiratory syndrome coronavirus 2 (SARS-CoV-2) was detected by polymerase chain reaction (PCR) test of a nasopharyngeal swab. Patients were determined to be obese (BMI > 30 kg/m^2^) or overweight (BMI 25–29 kg/m^2^) according to the World Health Organization (WHO) classification [1].

Our tertiary ICU has a standard capacity for 32 patients, which was expanded to 62 beds during the COVID-19 pandemic. An ECMO rescue team is available 24 h per day to cannulate and transport a patient from their original hospital to our Extracorporeal Life Support (ECLS) center.

The data were collected from April 1, 2020, to May 31, 2022, matching the period with the greatest number of ICU admissions of COVID-19 patients in Portugal [9].

The study was approved by Coimbra Hospital and University Centre’s Ethics Committee (n° OBS.SF.202–2022).

### Data collection and outcomes

The data were gathered retrospectively through patient medical records consultation.

The following patient characteristics were collected: age, sex, BMI, comorbidities, Charlson score, Acute Physiology and Chronic Health Evaluation (APACHE II) score, Sequential Organ Function (SOFA) score before ECMO initiation, invasive mechanical ventilation duration prior to ECMO, and last PaO_2_/FiO_2_ before ECMO. We identified patients who were transported from other hospitals on ECMO.

The main outcomes were 60-day and 180-day survival after ICU admission. We also evaluated the duration of mechanical ventilation, duration of ECMO, rate of successful weaning from ECMO, length of ICU stay, and length of hospital stay.

We recorded the type of anticoagulation used during ECMO, the need for blood product transfusion, the use of prone position on ECMO, and complications related to the ICU stay or to ECMO: ECMO cannulation complications, bleeding, thrombosis, ischemia, associated cannula infections, bacteremia, and ventilator-associated pneumonia.

### Statistical analysis

The data are expressed as medians and interquartile ranges (IQRs) or as numbers and percentages (%). Patient characteristics were compared according to 180-day survival after ICU admission. Normality in continuous variables was assessed by histogram visualization and the Shapiro‒Wilk test, those with a normal distribution were compared using Student’s t-test (parametric test), and those without a normal distribution were compared using the Mann‒Whitney *U* test (nonparametric test). Categorical variables were compared using the chi-square test or Fisher’s exact test. Univariate logistic regression analysis was performed to assess differences in 180-day survival.

Statistical significance was set at *p* < 0.05. Statistical analysis was performed using SPSS statistical software (version 20.0).

## Results

Among a total of 45 COVID-19 patients supported with VV-ECMO during the abovementioned period, 41 were included due to obesity or overweight.

Regarding specific COVID-19 pneumonia treatment, 38 (92.7%) patients were treated with dexamethasone, as recommended in national guidelines [10]; the remaining 3 (7.3%) patients were treated with hydroxychloroquine during the first COVID-19 outbreak, according to the therapeutic indications at that time.

The patients’ median age was 55 (45–60) years, and 70.7% were male. At baseline, the median BMI was 36 (31–42.5) kg/m^2^ (minimum 26 kg/m^2^; maximum 59 kg/m^2^), and 39% of patients had class III obesity (BMI ≥ 40 kg/m^2^). Most patients (73.2%) were transported from other hospitals to our extracorporeal life support (ECLS) center on ECMO.

The patients had a median of 3 (IQR 2–4) days of invasive ventilation before ECMO and a median PaO_2_/FiO_2_ ratio of 74 (IQR 59–100) mmHg before ECMO initiation. The median Charlson Comorbidity Index score was 1 (IQR 0–2), and the most common comorbidities were hypertension (51.2%), dyslipidemia (34.1%), and diabetes (17.1%).

The median ECMO duration was 19 (IQR 11–34) days (minimum 1 day, maximum 57 days), the invasive ventilation duration was 30 (IQR 19–50) days, and 63.4% of patients were weaned from ECMO. One of the patients underwent a second run on ECMO. The median length of stay in the ICU was 32 (IQR 18–45) days and that in the hospital was 48 (IQR 30–60) days. The 60-day and 180-day mortality rates were both 41.5% (17 patients), and the hospital mortality rate was 39% (16 patients) (see Table 1).

**Table 1:** Cohort outcomes

Outcome	*N* = 41 patients
Weaning from ECMO, *n* (%)	26 (63.4)
Hospital mortality, *n* (%)	16 (39)
180-days mortality, *n* (%)	17 (41.5)
ICU length of stay (days)	32 (18–45)
Hospital length of stay (days)	48 (30–60)

The data are expressed as medians (interquartile ranges) or numbers (percentages, %)

*ECMO* extracorporeal membrane oxygenation, *ICU* intensive care unit

Patient characteristics and comparisons according to survival on day 180 are presented in Table 2.

**Table 2:** Patient characteristics and comparisons according to 180-day survival

	**All (*n* = 41)**	**Survivors at D180 (*n* = 24)**	**Non survivors at D180 (*n* = 17)**	***p* Value**
**Baseline patient’s characteristics**				
Age (years)	55 (45–60)	48 (42–57)	56 (55–64)	< 0.001
Male sex, *n* (%)	29 (70.7)	13 (54.2)	16 (94.1)	0.006
BMI (kg/m^2^)	36 (31–42.5)	41 (32.3–49.5)	32 (28.5–37.5)	0.001
WHO BMI classification, *n* (%)				0.059
Overweight (25–29.9 kg/m^2^)	8 (19.5)	2 (8.3)	6 (35.3)	
Class I obesity (30–34.9 kg/m^2^)	9 (22)	5 (20.8)	4 (23.5)	
Class II obesity (35–39.9 kg/m^2^)	8 (19.5)	4 (16.7)	4 (23.5)	
Class III obesity (≥ 40 kg/m^2^)	16 (39)	13 (54.2)	3 (17.6)	
MV duration before ECMO (days)	3 (2–4)	2 (1–3)	4 (2–6)	0.033
PaO2/FiO2 before ECMO (mmHg)	74 (59–100)	75 (56–94)	72 (64–102)	0.616
Charlson comorbidity index score	1 (0–2)	0 (0–2)	2 (1–3)	0.006
Interhospital transfer on ECMO, n (%)	30 (73.2)	16 (66.7)	14 (82.4)	0.141
**Severity scores**				
APACHE II	14 (11–18)	13 (10–15)	17 (11–22)	0.065
Last SOFA prior to ECMO	8 (6–9)	7 (5–9)	8 (8–10)	0.01

The data are expressed as medians (interquartile ranges) or numbers (percentages, %)

*BMI* body mass index (kg/m^2^), *WHO* World Health Organization, *MV* mechanical ventilation, *ECMO* extracorporeal membrane oxygenation, *APACHE II* Acute Physiology and Chronic Health Evaluation, *SOFA sequential organ failure assessment*

Regarding therapeutic measures, anticoagulation therapy with heparin was used in 97.6% of patients, bivalirudin in 9.8%, fondaparinux in 4.9%, and argatroban in 2.4%. All patients were transfused with blood products during ECMO support (detailed description in Table 3). Fifty one point two percent of patients were placed in the prone position on ECMO, of which 2 (9%) had excess weight, 4 (19%) were categorized as having obesity Class I, 4 (19%) had obesity Class II, and 9 (43%) had obesity Class III. ICU and ECMO associated complications are described in Table 3.

**Table 3:** Therapeutic measures, complications, and outcomes related to ECMO support and comparison according to 180-day survival

	**All (*n* = 41)**	**Survivors at D180 (*n* = 24)**	**Non-survivors at D180 (*n* = 17)**	***p* Value**
**Complications during ECMO, *n* (%)**				
*Infection*				
Ventilatory-acquired pneumonia	32 (78)	18 (75)	14 (82.4)	0.711
Bacteriemia	18 (43.9)	9 (37.5)	9 (52.9)	0.285
Cannula site infection	7 (17.1)	5 (20.8)	2 (11.8)	0.679
*ECMO cannulation complication*	9 (22)	2 (8.3)	7 (41.2)	0.021
*Ischemia*	0	0	0	
*Venous thrombosis*	10 (24.4)	7 (29.2)	3 (17.6)	0.48
*Bleeding*				
Cannula site	28 (68.3)	18 (75)	10 (58.8)	0.273
Other intravascular catheter site	25 (61)	17 (70.8)	8 (47.1)	0.124
Intracranial	2 (4.9)	0	2 (11.8)	0.166
Gastrointestinal	3 (7.3)	1 (4.2)	2 (11.8)	0.56
Hematuria	29 (70.7)	19 (79.2)	10 (58.8)	0.184
Hemoptysis	34 (82.9)	20 (83.3)	14 (82.4)	1.0
Naso and oropharynx	32 (78)	17 (70.8)	15 (88.2)	0.262
Soft tissue/skeletal muscle	10 (24.4)	4 (16.7)	6 (35.3)	0.27
**Therapeutics during ECMO**				
*Anticoagulation, n (%)*				
Heparin	40 (97.6)	24 (100)	16 (94.1)	0.415
Bivalirudin	4 (9.8)	2 (8.3)	2 (11.8)	1.0
Fondaparinux	2 (4.9)	1 (4.2)	1 (5.9)	1.0
Argatroban	1 (2.4)	1 (4.2)	0	1.0
*Blood products transfusion*				
Erythrocyte concentrate (U)	10 (4–21)	8.5 (2.3–18.8)	12 (6–27.5)	0.172
Platelets (U)	2 (0–4.5)	1 (0–4)	2 (0.5–7.5)	0.331
Plasma (U)	0 (0–0)	0	0 (0–0.5)	0.059
Fibrinogen (g)	2 (0–8)	2 (0–7)	2 (0–8)	0.911
*Prone position on ECMO, n (%)*	21 (51.2)	12 (50)	9 (52.9)	1.0
**Outcomes**				
ECMO duration (days)	19 (10.5–34)	16.5 (8–34)	27 (14.5–34)	0.491
MV duration (days)	30 (19–49.5)	29.5 (19.5–50)	32 (18.5–46)	0.604

The data are expressed as medians (interquartile ranges) or numbers (percentages, %)

*ECMO* extracorporeal membrane oxygenation, *MV* mechanical ventilation

The predictors of 180-day survival in this population of obese and overweight patients who were supported with ECMO were significantly higher BMI, younger age, female sex, lowest Charlson Comorbidity Index score, shorter duration of mechanical ventilation prior to ECMO, and fewer complications at ECMO cannulation (Table 4). We performed a univariate analysis of 180-day survival within the four groups of BMI, and this analysis did not show any impact on survival (Table 5).

**Table 4:** Univariate analysis of predictors of 180-day survival

Variables	Odds ratio	95% CI	*p* Value
Younger age	0.858	0.774–0.953	0.004
Higher BMI	1.157	1.038–1.291	0.009
Female	0.074	0.008–0.650	0.019
Lowest Charlson score	0.422	0.216–0.825	0.012
Shorter MV duration before ECMO	0.612	0.401–0.933	0.022
Fewer ECMO cannulation complications	0.13	0.023–0.740	0.022

The data are expressed as medians (interquartile ranges) or numbers (percentages, %)

*BMI* body mass index (kg/m^2^), *MV* mechanical ventilation, *ECMO* extracorporeal membrane oxygenation

**Table 5:** Univariate analysis of predictors of 180-day survival in different groups of BMI

Variables	Odds ratio	95% CI	*p* Value
Overweight (BMI 25–29.9 kg/m^2^)	0.442	0.259–22.025	0.442
Class I obesity (BMI 30–34.9 kg/m^2^)	1.089	0.403–2.944	0.867
Class II obesity (BMI 35–39.9 kg/m^2^)	0.88	0.431–1.798	0.72
Class III obesity (BMI ≥ 40 kg/m^2^)	1.873	0.665–5.280	0.235

*BMI* body mass index (kg/m^2^)

## Discussion

In this single-center cohort with 41 obese and overweight COVID-19 patients supported with VV-ECMO because of severe ARDS, the 180-day mortality rate was 41.5%. Most published studies have described the same mortality rate as ours in COVID-19 patients supported with VV-ECMO. These rates vary from 38 to 58% [11–14].

The predictor factors for 180-day survival were a higher BMI, younger age, female sex, a lowest Charlson Comorbidity Index score, a shorter duration of mechanical ventilation prior to ECMO, and fewer complications at ECMO cannulation.

Several prognostic factors for survival in ECMO patients are known in the literature and are used to decide on extracorporeal membrane oxygenation initiation [15]. Age is an independent risk factor, present in most survival predictive scores [16, 17], and younger age is associated with greater survival, as observed in the obese and overweight patients in our cohort. A recent meta-analysis also described that male sex was probably associated with increased mortality in COVID-19 patients receiving VV-ECMO [18]. Alongside the VV-ECMO recommendations for COVID-19-related ARDS and other published literature, a better outcome was achieved in patients with a shorter mechanical ventilation time prior to ECMO [14, 19]. Fewer comorbidities, represented by a lower Charlson Comorbidity Index score, were related to better survival. The ECMO team has the responsibility to carefully select the patients who might benefit the most from ECLS, and in this cohort, the median Charlson comorbidity index was mild.

In our cohort, a higher BMI was also associated with 180-day survival, even though obesity is associated with an increased risk of death in patients with SARS-CoV-2 infection [20]. Furthermore, it is a risk factor for acute kidney injury in critical care patients with ARDS [21] and is associated with an increased prevalence of chronic heart and kidney disease [3].

Although obesity is a risk factor for severe disease, an association between obesity and improved outcomes has been previously reported, both in general critical care patients and those receiving ECLS [2, 22–25]. This phenomenon has been described as the “obesity paradox” [26]. Suggested explanations for this protective effect are the presence of more nutritional reserves and the immunomodulatory effects of substances secreted by fat cells. Additionally, in critical illness, adipose tissue adapts by increasing the storage of circulating lipids, which might lower insulin resistance and the harmful effects of serum glucose and lipids during the catabolic phase [27]. During ECMO support, a greater survival rate in obese patients could be explained by the presence of less parenchymal lung disease at the time of ventilation failure in obese patients than in normal weight patients due to altered respiratory mechanics in obese patients [28]. Recently, a retrospective analysis of the Extracorporeal Life Support Organisation (ELSO) Registry revealed a lower mortality risk among patients with a BMI > 35 kg/m^2^, with no upper limit indicating the futility of ECMO treatment identified [25]. In our study, a univariate analysis of 180-day survival within the four groups of BMI could not identify an obesity class with more impact in survival, possibly due to the small sample of patients in each BMI group.

However, there are contradictory results regarding the impact of obesity on critically ill patients, and some studies have not shown any survival advantage in obese patients [29, 30]. Limitations associated with observational and retrospective analyses could be the reason for the different results.

Peripheral cannulation access in obese patients can be challenging, even though no significant complications are found in obese patients receiving VV-ECMO compared to normal weight patients [31, 32]. However, protocols should be implemented to decrease cannulation complications since they can be associated with increased mortality, as shown in our cohort.

According to a 2020 data analysis of adult COVID-19 patients from the ELSO Registry, although 70% of patients were transferred from another hospital to an ELSO center, only 47% of them were transported while receiving ECMO support [11].We had a high percentage of overweight and obese patients who were transported on ECMO from other hospitals (73.2%), with no impact on their mortality. Obesity is not a contraindication for transport on ECMO [33] and should be considered if it is beneficial to the patient.

Because this is a retrospective and monocentric study, there are limitations regarding the type of data available for analysis and the capacity to apply the knowledge in other ICUs. The cohort also had a small sample size, preventing us from conducting a multivariable analysis, which lowered the power of the results. However, the results suggest several predicting factors for survival to be considered when initiating ECMO in obese patients, and we believe that these factors should be further explored in larger cohorts and randomized controlled trials. Future studies should address the BMI cutoff value that best correlated with survival.

## Conclusions

In this retrospective cohort of critical COVID-19 obese adult patients supported by VV-ECMO, a higher BMI, younger age, and female sex were associated with greater 180-day survival. A shorter invasive ventilation time prior to ECMO and fewer complications at ECMO cannulation were also associated with increased survival.

## Data Availability

The data that support the findings of this study are available from the authors, but restrictions apply to the availability of these data, which were used under authorisation from the Coimbra University Hospital Centre for the current study and are not publicly available. However, the data are available from the authors upon reasonable request and with permission from the Coimbra University Hospital Centre.

## Abbreviations

APACHE II: Acute Physiology and Chronic Health Evaluation II
ARDS: Acute respiratory distress syndrome
BMI: Body mass index
CI: Confidence interval
ECLS: Extracorporeal life support
ECMO: Extracorporeal membrane oxygenation
ELSO: Extracorporeal Life Support Organisation
ICU: Intensive care unit
IQR: Interquartile range
MV: Mechanical ventilation
OR: Odds ratio
PCR: Polymerase chain reaction
SARS-CoV-2: Severe acute respiratory syndrome coronavirus 2
SOFA: Sequential organ function
VV-ECMO: Venovenous extracorporeal membrane oxygenation
WHO: World Health Organization
